# Lung stereotactic body radiation therapy: personalized PTV margins according to tumor location and number of four-dimensional CT scans

**DOI:** 10.1186/s13014-021-01973-5

**Published:** 2022-01-10

**Authors:** Pierre Trémolières, Ana Gonzalez-Moya, Amaury Paumier, Martine Mege, Julien Blanchecotte, Christelle Theotime, Damien Autret, Stéphane Dufreneix

**Affiliations:** 1 Department of Radiation Oncology, Institut de Cancérologie de L’Ouest Angers, 15 Rue A Boquel, 49055 Angers Cedex 02, France; 2 Department of Medical Physics, Institut de Cancérologie de L’Ouest Angers, 15 Rue A Boquel, 49055 Angers Cedex 02, France

**Keywords:** Lung tumors, Stereotactic body radiation therapy, PTV margin, Motion study, Four dimensional computed tomography

## Abstract

**Objectives:**

To characterise the motion of pulmonary tumours during stereotactic body radiation therapy (SBRT) and to evaluate different margins when creating the planning target volume (PTV) on a single 4D CT scan (4DCT).

**Methods:**

We conducted a retrospective single-site analysis on 30 patients undergoing lung SBRT. Two 4DCTs (4DCT1 and 4DCT2) were performed on all patients. First, motion was recorded for each 4DCT in anterior–posterior (AP), superior-inferior (SI) and rightleft (RL) directions. Then, we used 3 different margins (3,4 and 5 mm) to create the PTV, from the internal target volume (ITV) of 4DCT1 only (PTV D1 + 3, PTV D1 + 4, PTV D1 + 5). We compared, using the Dice coefficient, the volumes of these 3 PTVs, to the PTV actually used for the treatment (PTV_ttt_). Finally, new treatment plans were calculated using only these 3 PTVs. We studied the ratio of the D2%, D50% and D98% between each new plan and the plan actually used for the treatment (D2% PTV_ttt_, D50% PTV_ttt_, D50% ITVttt D98% PTV_ttt_).

**Results:**

30 lesions were studied. The greatest motion was observed in the SI axis (8.8 ± 6.6 [0.4–25.8] mm). The Dice index was higher when comparing PTVttt to PTV D1 + 4 mm (0.89 ± 0.04 [0.82–0.98]). Large differences were observed when comparing plans relative to PTVttt and PTV D1 + 3 for D98% PTV_ttt_ (0.85 ± 0.24 [0.19–1.00]).

and also for D98% ITV_ttt_ (0.93 ± 0.12 [0.4–1.0]).D98% PTV_ttt_ (0.85 ± 0.24 [0.19–1.00], *p* value = 0.003) was statistically different when comparing plans relative to PTV_ttt_ and PTV D1 + 3. No stastistically differences were observed when comparing plans relative to PTV_ttt_ and PTV D1 + 4. A difference greater than 10% relative to D98% PTV_ttt_ was found for only in one UL lesion, located under the carina.

**Conclusion:**

A single 4DCT appears feasible for upper lobe lesions located above the carina, using a 4-mm margin to generate the PTV.

**Advance in knowledge:**

Propostion of a personalized SBRT treatment (number of 4DCT, margins) according to tumor location (above or under the carina).

## Background

Stereotactic body radiation therapy (SBRT) is recommended for inoperable stage I primary lung cancers, as well as for the treatment of pulmonary metastases [1, 2]. In lung SBRT, due to the low number of fractions and high dose gradient, it is essential to limit as far as possible uncertainty linked to respiratory motion [3]. Several methods are available to treat mobile targets including gating [4], tracking [5] and free-breathing treatments [6]. Free-breathing treatments ensure a high degree of coverage of the target volume, satisfactory sparing of healthy tissues and are far easier to implement than gating or tracking [7]. To take into account tumour motion, it is recommended that a four-dimensional computed tomography (4DCT) is used to plan the lung SBRT [8]. The 4DCT enhances the evaluation of tumour motion by delineating the tumour over several phases of the respiratory cycle, thus creating a personalised volume for each patient, the internal target volume (ITV), and improving coverage of the target-volume [9]. Tumour motion is location-dependent, with greater motion generally observed in lower lobe tumours [10, 11]. Motion is also specific to each patient and may change over time [12]. Motion reproductibility may not be taken into account when a single 4DCT is used, resulting in potential differences in the coverage of target-volumes. A single 4DCT seems reliable for a majority of patients, with nevertheless uncertainties increased for lower lobe tumors [13]. However, there are no recommendations on the number of 4DCTs required [14]. Free-breathing treatments are used in our institution and two 4DCTs are systematically performed for all lung SBRT treatments. This study was carried out to evaluate a personalized planning target volume (PTV) margin according to the tumor location, if only one 4DCT was performed. Motion of pulmonary tumours was also studied.

## Methods

A retrospective single-site analysis was conducted at Institut de Cancérologie de l’Ouest in Angers on 30 patients undergoing SBRT for primary or secondary lung tumours, in the upper lobes (UL) or lower lobes (LL).

### Planning CT

4DCTs were performed using a GE Lightspeed CT-scan for 15 patients (slice thickness: 2.5 mm) then, due to a change in equipment, Siemens Big Bore CT-scan for the other 15 patients (slice thickness: 1 mm). For the acquisition of the breathing signal, a Varian Real-time Position Management (RPM) was used. Acquisitions were conducted in a free-breathing mode, without fiducial markers. Two 4DCTs (4DCT1 and 4DCT2) were performed on all patients, on two different days (at 24- or 48-h intervals). Patients received no specific breathing instructions (no coaching). Patients were placed in an Orfit All-In-One (AIO) position, either with their arms raised without mask, or arms parallel to their body with a five-point mask encompassing the head and shoulders. Images were separated into 10 phases (0% to 90%) which divided the breathing cycle equally. Phase 0% corresponded to maximum inspiration and phase 50%, approximately maximum expiration. An “average” scan was calculated representing an average image of these 10 phases.

### Contouring

Gross Tumour Volume (GTV) was delineated on each phase of the breathing cycle, for 4DCT 1 and 2. Internal Target Volume (ITV) was defined as the union of the 10 GTVs of each phase of the cycle. Thus, two different ITVs were obtained, one from 4DCT1 (ITV_D1_) and one from 4DCT2 (ITV_D2_). The two 4DCTs were registered automatically by bone registration, then manually by the radiation oncologist for registration to the lesion. A total ITV (ITV_ttt_) was then determined, representing the sum of ITV_D1_ and ITV_D2_. PTV_ttt_ was defined from ITV_ttt,_ with a 3-mm margin in all directions. Contouring and registration were analysed based on the ones established by the radiation oncologist during the treatment planning. Four different radiation oncologists experienced in lung stereotactic techniques (who had used the technique since 2008 at our institution) treated the patients included in the study.

### Motion study

Tumour size was defined using the largest diameter measured on one phase in the axial plane. The motion of lesions defined as the maximum distance travelled by the centre of mass of the lesion over the 10 phases were recorded for each 4DCT in anterior–posterior (AP), superior-inferior (SI) and right-left (RL) directions. The average motion of the lesion in these three directions was defined as the average motion on the two 4DCTs performed. The difference in motion of the same lesion between the two 4DCTs was also analysed in the three directions.

### Study of volumes

ITV_D1_ and ITV_ttt_ volumes were compared. We estimated whether a greater PTV margin on 4DCT1 could compensate for the lack of additional information derived from the 4DCT2, three different margins relative to ITV_D1_ should be used: i.e. the usual 3-mm margin, a 4-mm margin and a 5-mm margin. The PTV volumes were compared by studying the ratio between the two volumes, taking the PTV_ttt_ as reference. A coverage measurement was also used, the Dice coefficient, for greater robustness. For two separate volumes, the Dice coefficient was defined by:

$$Dice = \frac{{2 \left( {Volume_{1} \cap Volume_{2} } \right)}}{{Volume_{1} + Volume_{2} }}$$

The result always ranged between 0 and 1. The closer the result to 1, the greater the comparability of the two volumes in terms of size and location.

### Dosimetry study

The Eclipse software (Varian Medical System) was used to define the treatment plans on a Novalis Trubeam STx (Varian Medical System) in X6 FFF. The prescribed dose was 50 Gy in five fractions of 10 Gy, on the 80% isodose. 100% of the prescribed dose should cover 98% of the PTV, with a D2% stated as 125%. A fixed beam stereotactic technique was used. In parallel to the dosimetry for the treatment plan based on PTV_ttt_, three new treatment plans were calculated using only 4DCT D1. The first plan was based on PTV_D1+3_ (ITV_D1_ + 3 mm), the second plan on PTV_D1+4_ (ITV_D1_ + 4 mm) and the third plan on PTV_D1+5_ (ITV_D1_ + 5 mm). Thus, plans based on PTV_D1+3_, PTV_D1+4_ and PTV _D1+5_ represented treatment plans delivered through the use of a single 4DCT, applying a 3-, 4- or 5-mm margin. Irradiation geometry (number of beams, arm angles, etc.) was identical for the three treatment plans, only the multileaf collimator was adjusted to comply with the related PTV. The AcurosXB (v13.7) algorithm was used for all calculations. The ratio of metrics was analysed taking as denominator the measurement reported for the PTV_ttt_ based plan. A ratio of 1 showed that the two treatment plans were equivalent relative to the PTV_ttt_ measurement, whereas a ratio smaller than 1 indicated an under-dosage of PTV_ttt_ if a single 4D-CT was being considered. The metrics studied were, in accordance with ICRU 91 recommendations [15], maximum dose (D2%_PTVttt_), median dose (D50%_PTVttt_) and minimum dose (D98%_PTVttt_) relative to PTV_ttt_, as well as the minimum dose (D98%_ITVttt_) and the median dose (D50%_ITVttt_) relative to ITV_ttt_.

The same presentation was used for all results in the next section: mean ± standard deviation [minimum value–maximum value] unit. Statistical significance was analysed using a Welch Two Sample t-test.

## Results

Of the 30 patients enrolled in the study, characteristics were comparable between location, origin and slice thickness (Table 1). The mean size of the lesions was 15.5 ± 6.5 [7–34] mm.

**Table 1:** Characteristics and motion of tumour lesions

Position	Size (mm)	LR motion (mm)		AP motion (mm)		SI motion (mm)	
		4DCT1	4DCT2	4DCT1	4DCT2	4DCT1	4DCT2
UL	13	1.2	1.3	1.5	1.4	8	5.5
LL	12	0.5	1	4	2.8	21.2	14.7
LL	8	1.4	1.7	3.3	2.4	1.3	1.2
LL	8	0.5	1	0.8	1.8	2.2	3.9
LL	9	0.9	1	0.9	0.9	14.7	12.9
LL	14	1.7	2.6	2.2	0.9	16.9	10.5
LL	10	1.6	0.9	3.4	4.2	12.2	13.9
UL	12	1.5	1.5	3.6	4.6	3.5	2.3
UL	7	0.2	0.5	0.1	0.7	0.3	0.5
LL	9	2.1	1.2	2.3	1.8	18.4	18.8
UL	18	2.9	2.3	1.7	1.7	1.6	2.4
LL	13	2.1	2.5	4.2	5.5	12.4	15.8
LL	20	2.5	1.7	2.2	2.7	10.8	11.1
UL	18	1.6	1.9	3	2	3.4	1.6
UL	25	1.7	1	10	4.7	11.8	5.7
UL	16	0.5	0.7	1.5	2.7	2.4	3
LL	20	0.8	1.6	2.4	1.9	11.4	10.6
UL	15	0.6	0.5	1	0.6	1	1.4
LL	16	1.6	2.5	2.3	4.9	15.6	24.1
UL	25	0.4	1.3	2.4	3.7	1.6	1.9
UL	10	2	1.4	6.6	4.4	5.6	6
UL	34	4.5	3.7	6.2	5.2	11.4	11.4
LL	13	0.7	1.2	3.6	4.2	8	9.9
LL	13	3.2	3.5	1.5	2.5	25.4	26.2
LL	12	1.5	1.9	3.9	2.4	14.4	8.8
LL	13	1.1	0.6	3	1.6	7.2	7
UL	21	1.1	0.8	1.8	1.4	3.6	2.5
UL	31	1	1.2	2.1	1.9	0.7	1
UL	17	0.6	1.3	2.9	3.2	9	10.3
UL	12	2.4	2.8	3.2	3.8	10	14.9

### Motion study

The greatest motion was observed in the SI axis (8.8 ± 6.6 [0.4–25.8] mm), with motion 2.6 times greater for LL lesions (12.7 ± 6.4 [1.3–25.8] mm) compared to UL lesions (4.8 ± 4.4 [0.4–12.5] mm). Differences in motion between the same lesions, on the two 4DCTs, were also greater on the SI axis (2.1 ± 2.4 [0.0–8.5] mm) with differences more marked for LL lesions (2.7 ± 2.7 [0.1–8.5] mm) than UL lesions (1.5 ± 1.8 [0.0–6.1] mm). Differences in motion exceeded 3 mm in 23% of patients, including two UL lesions (13%) and five LL lesions (33%). In the LR axis, differences in motion were systematically smaller than 1 mm and, in the AP axis, a motion difference greater than 3 mm was observed in only one lesion.

### Study of volumes

The ITV_D1_ was on average 13% lower than the ITV_ttt_ resulting from a union of ITV_D1_ and ITV_D2_, with a ratio of 0.87 ± 0.13 [0.53–1]. No major difference were observed between UL lesions (0.89 ± 0.14 [0.53–1]) and LL lesions (0.85 ± 0.12 [0.62–1]). Similar results were observed for the PTV_D1+3 mm_ (0.87 ± 0.12 [0.59–1]).

PTV_D1+4 mm_ tended to be larger than the PTV_ttt_ (1.11 ± 0.19 [0.71–1.45]). Finally, PTV_D1+5 mm_ volume was greater than the PTV_ttt_ with an average 37% overestimation (1.37 ± 0.27 [0.87–1.94]).

**Fig. 1:**
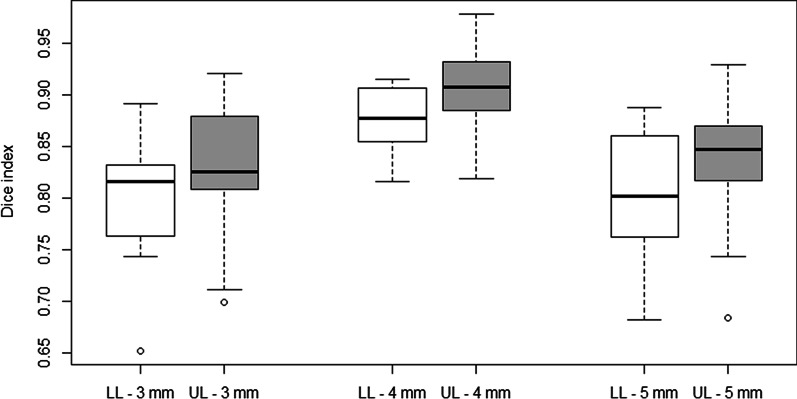
Dice indices between PTVttt and PTV created using three different margins on ITV_D1_

The highest Dice index was found when comparing PTV_ttt_ to PTV_D1+4 mm_ (0.89 ± 0.04 [0.82–0.98]) (Fig. 1). Dice indices allowing a comparison of PTV_ttt_ and PTV_D1 + 3 mm_ volumes as well as PTV_ttt_ and PTV_D1 + 5 mm_ were comparable (0.81 ± 0.06 [0.65–0.92] and 0.82 ± 0.06 [0.68–0.93] respectively).

### Dosimetry study

**Fig. 2:**
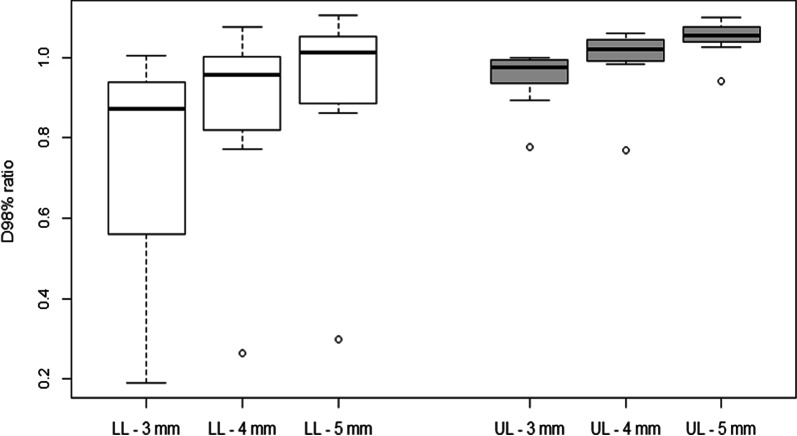
D_98%_ to PTV_ttt_ ratio for dosimetry calculated only relative to PTV_D1+3,_ PTV_D1+4_ and PTV_D1+5_

**Fig. 3:**
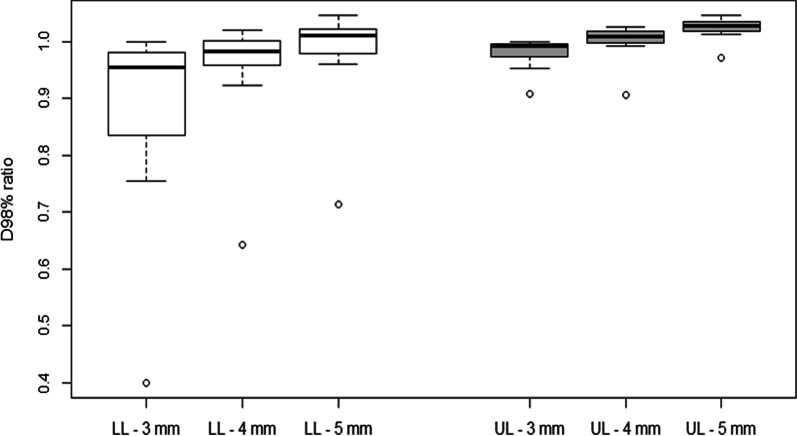
D_98%_ to ITV_ttt_ ratio for dosimetry calculated only relative to PTV_D1+3,_ PTV_D1+4_ and PTV_D1+5_

Results for the D98%_PTVttt_ and D98%_ITVttt_ ratios are shown in Figs. 2 and 3.

A comparison of plans relative to PTV_ttt_ and PTV_D1+3_ (ratio between metrics of these two plans) did not reveal any statistically significant differences in relation to D2%_PTVttt_ (1.00 ± 0.01 [0.97–1.00], *p* value = 0.12), D50%_ITVttt_ (0.99 ± 0.01 [0.97–1.00], *p* value = 0.47) and D50%_PTVttt_ (0.98 ± 0.02 [0.93–1.00], *p* value = 0.37). The difference relative to D98%_PTVttt_ indicated an average 15% under-dosage of the PTV_ttt_ when dosimetry was calculated using a single 4DCT and a 3-mm margin applied. This difference was statistically significant (0.85 ± 0.24 [0.19–1.00], *p* value = 0.003). Greater differences were observed between LL lesions (0.75 ± 0.30 [0.19–1.00], *p* value = 0.007) in relation to which there was a greater under-dosage versus UL lesions (0.95 ± 0.06 [0.78–1.00], *p* value = 0.39). A difference of more than 10% on D98%_PTVttt_ was observed in eight LL lesions (53%) and only in two UL lesions (13%). Regarding the D98%_ITVttt_, differences were statistically significant for UL lesions (0.98 ± 0.03 [0.91–1.00], p value = 0.02) and for LL lesions (0.89 ± 0.16 [0.4–1.00], *p* value = 0.02).

A comparison of plans relative to PTV_ttt_ and PTV_D1+4_ did not reveal any statistically significant difference in relation to D2%_PTVttt_ (1.00 ± 0.00 [0.98–1.00], *p* value = 0.09), D50%_ITVttt_ (1.00 ± 0.01 [0.98–1.02], *p* value = 0.12) and D50%_PTVttt_ (1.00 ± 0.01 [0.96–1.03], *p* value = 0.66). The difference relative to D98%_PTVttt_ indicated an average 5% under-dosage of the PTV_ttt_ when dosimetry was calculated using a single 4DCT and applying a 4-mm margin (0.95 ± 0.16 [0.27–1.08], *p* value = 0.56). Although non-significant, the under-dosage was greater for LL lesions (0.90 ± 0.20 [0.27–1.08], *p* value = 0.62) and the D98%_PTVttt_ measurement of UL lesions was on average identical for both plans (1.01 ± 0.07 [0.77–1.06], *p* value = 0.31). A difference greater than 10% relative to D98%_PTVttt_ was observed in five LL lesions (33%) and only in one UL lesion (7%). Regarding the D98%_ITVttt_, differences were not statistically significant for UL lesions (1.00 ± 0.03 [0.91–1.03], *p* value = 0.72) and LL lesions (0.96 ± 0.09 [0.64–1.02], *p* value = 0.15).

A comparison of plans relative to PTV_ttt_ and PTV_D1+5_ revealed small statistically significant difference in relation to D2%_PTVttt_ (1.00 ± 0.00 [0.99–1.01], *p* value = 0.001), D50%_ITVttt_ (1.01 ± 0.01 [0.99–1.02], *p* value = 1E−08) and D50%_PTVttt_ (1.02 ± 0.01 [0.99–1.04], *p* value = 1E−09). The difference relative to D98%_PTVttt_ indicated a similar coverage of the PTV_ttt_ when dosimetry was calculated using a single 4DCT and applying a 5-mm margin (1.00 ± 0.15 [0.30–1.10], *p* value = 0.98). A non-significant 5% under-dosage was observed for LL lesions (0.95 ± 0.20 [0.30–1.10], *p* value = 0.33) and a significant 5% over-dosage was observed for UL lesions (1.05 ± 0.04 [0.94–1.10], *p* value = 0.0002). Regarding the D98%_ITVttt_, differences were not statistically significant for LL lesions (0.99 ± 0.08 [0.71–1.05], *p* value = 0.51) and significant for UL lesions (1.02 ± 0.02 [0.97–1.05], *p* value = 0.0002).

## Discussion

The motion study reported results comparable with data from the literature with significant motion observed primarily in the SI axis and to a greater extent in LL lesions (10–12). It also showed that tumour movements are not reproducible from one 4DCT to the other with a mean difference in motion of 2.1 mm. The difference in motion was greater than 3 mm in 33% of LL lesions and 13% of UL lesions, reflecting a much greater difference in motion from day to day that would not be covered by applying a 3-mm margin.

PTV generated from 4DCT1 using a 3 mm margin generates a smaller volume than the PTV volume resulting from combining ITVs from 4DCT1 and 4DCT2 with a 3 mm margin. To allow a single 4DCT to be used when planning treatment, one solution would be to increase the margin when establishing the PTV based on the ITV. Using a 4-mm margin allows the Dice index to be maximised.

**Fig. 4:**
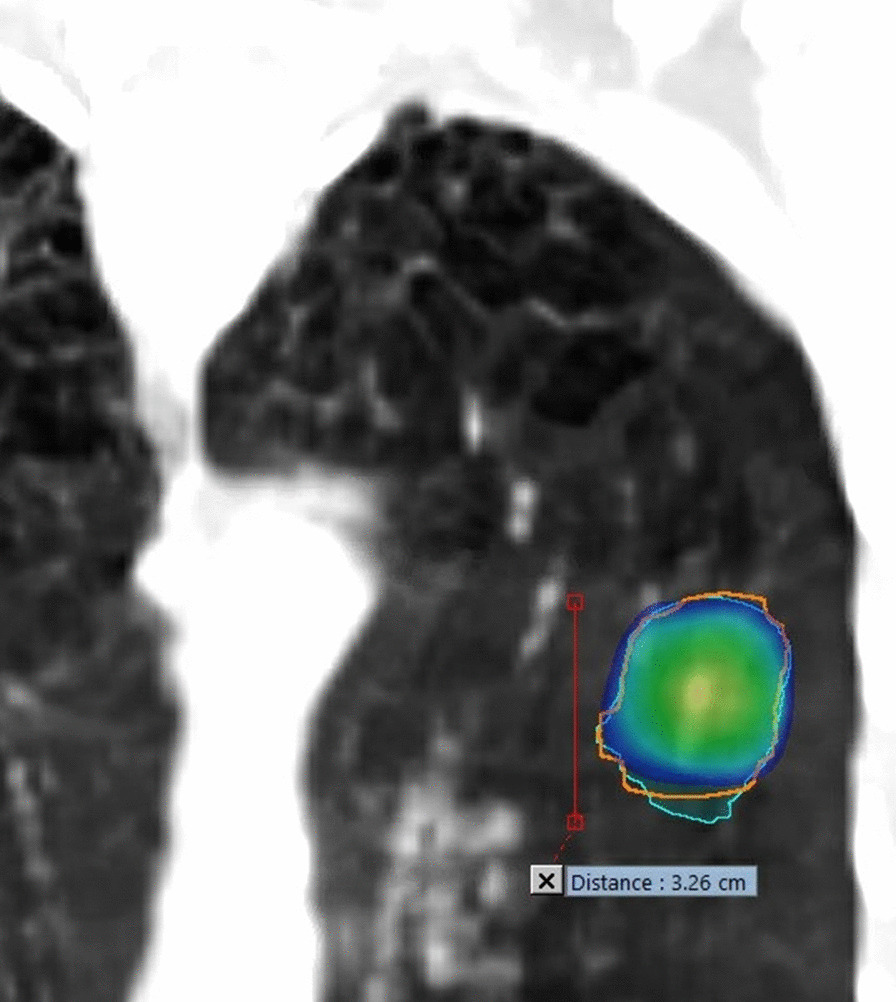
Front view of the left UL lesion under the carina. In blue PTVttt and in orange PTV_D1+4_. In blue and green sphere, 50 Gy isodose of the plan based on PTV_D1+4_

In terms of dosimetry, performing a single 4DCT for lung SBRT and the use of a 3-mm margin when creating the PTV induce significant differences in relation to D98%_PTVttt_ and D98%_ITVttt_ for both UL and LL lesions. This shows that a single 4DCT associated to a 3 mm margin does not properly take into account the non-reproducibility of tumor motion. Despite a rather satisfactory Dice index between volumes of PTV_ttt_ and PTV_D1+4_, the use of a single 4D scan and a 4 mm margin produced a D98%_PTVttt_ underestimation of more than 10% in five LL lesions and in only one UL lesion. This lesion was located in the lingula, adhering to the fissure and located under the carina (Fig. 4). For the other UL lesions, D98%_PTVttt_ and D98%_ITVttt_ were similar (difference < 6% and < 3% respectively) for the two dosimetry calculations. The use of a single 4D scan and a 5 mm margin produced larger PTV volumes compared to the PTV_ttt_ resulting in a global over-dosage of the PTV_ttt_, especially for UL tumors. There was still an under-dosage of the PTV_ttt_ for LL tumors and for the lesion located in the lingula. A larger margin does not compensate for differences in motion between the two 4DCTs, since the motion of lesions is greater along the SI axis when the margin is isotropic.

The upper lobe/lower lobe dichotomy is convenient but may be too simplistic, especially for the left lung, as the upper lobe descends forwards to the diaphragmatic cupola. The study of Sörnsen de Koste et al*.* showed that “supra-diaphragmatic” lesions (i.e. located 3 cm from the diaphragmatic cupola) were the most mobile, whether located in the upper or lower lobe. “Caudal” lesions (i.e. located under the carina) were also more mobile than lesions located above the carina [16]. If the carina is used as the lower limit for UL lesions, the dichotomy can be refined and lesions with more similar motions grouped together.

In our department, it is now standard practice to perform a single 4DCT for UL lesions located above the carina, using a 4-mm margin based on the ITV to generate the PTV. For UL lesions located under the carina and for LL lesions, we still use two 4DCTs and a 3-mm margin based on the results found in this study.

A recently published study from Khadige et al*.* studied 50 patients treated with SBRT for lung lesions [17]. They performed three 4DCTs and also used the RPM system. However, they used the Maximum Intensity Projection (MIP) for contouring (with a 5-mm margin to obtain the PTV). ITV was checked during the 0% and 50% phases. Although the generation of ITVs from the MIP may seem satisfactory [18], there is a risk of underestimating the volume in highly mobile lesions [19, 20]. The study compared volumes from ITVs obtained on three 4DCTs as well as those delineated on CBCTs during treatment. Mean volumes for both UL and LL lesions were statistically comparable. Thus, they decided to use a single 4D CT for thoracic SBRT treatments, regardless of the location of the lesion.

Guckenberger et al*.* evaluated whether a single 4DCT was reliable for lung SBRT. They performed 4 repeated 4DCT every 10 min on 10 patients (and 14 lesions) immobilized in a stereotactic body frame, the same day. For 10 lesions (71%) differences in motion (peak to peak tumor motion) was stable within ± 2 mm. For 4 lesions (29%), differences in motion were greater than 3 mm, with a difference of 11 mm for a patient with a forced expiratory volume in 1 s less that 1 L. Drifts from 3 to 5 mm were observed for a majority of the lower lobes lesions. Authors concluded that treatment planning based on a single 4DCT is reliable, but uncertainties remains for patients with poor pulmonary function and lower lobe tumors [13].

A study by van der Geld et al*.* showed that a 80% isodose coverage exceeded 90% in two PTVs obtained from two 4DCTs. In one patient out of the 26, the 80% isodose covered only 82.5% of PTV, with part of the volume only covered by the 20% of the isodose. It was a LL lesion with a motion difference of 3.7 mm but a volume difference of 19%. The authors considered a 90% coverage by the 80% isodose as acceptable. They justified this tolerance given that the tumour was only present in the under-dosed area 10–20% of the time. The two 4DCTs were also performed consecutively on the same day, which probably underestimates the potential difference in motion from day to day [14].

The limitations of our study are first and foremost its retrospective nature.

It should also be noted that the manual registration performed by the radiation oncologist between the two 4DCTs inherently induces uncertainty. However, an automatic registration (“soft tissue” or “bone”) appears to be unsatisfactory given the precision required by the stereotactic technique, and a manual intervention to refine the registration of the two targets seems essential. Contouring and registrations were performed by different radiation oncologists (for a given patient these two steps were performed by the same radiation oncologist). This produces a limitation in terms of reproducibility between observers, but all radiation oncologists were highly experienced in the technique.

Margins of 3 to 5 mm are recommended in the literature. The most commonly used PTV margin is 5 mm [21–23]. We decreased it to 3 mm given that two 4DCTs and a repositioning CBCT were performed at the start and mid-treatment, at each session. In our cohort, we showed that in free-breathing conditions, a margin of 5 mm did not compensate for differences in motion in all patients between two 4D scans and could even result in excessive irradiation of healthy tissues with no dosimetric gain in terms of PTV coverage. The small number of patients studied may be a limitation from a statistical perspective, but consistent with the literature. Indeed, articles on this subject generally involve between 10 and 30 patients [12]. With a difference of slice thickness between the two scanners used in this study of 1.5 mm, the tumor motion in the SI direction may also contain some uncertainty.

Finally, it is reminded that the goal of a PTV margin is not to take account of differences in motion, which is the role of the ITV. However, with only one 4DCT, there is an uncertainty about the reproducibility of the movement which cannot be taken into account. Also, it was shown a significant under-dosage of the ITV_ttt_ (up to 60% for LL and 9% for UL) with a 3 mm margin associated to a single 4DCT, reduced to 3% for UL above the carina considering a 4 mm margin.

In the future, it could be worthwhile to conduct the same dosimetric study comparing ITVs delineated on CBCTs performed during treatment versus ITVs delineated on 4DCT(s). For example, Purdie et al. reported a difference in motion (of 6 and 10 mm) in 2 out of 12 patients, between the motion observed on a 4DCT and the motion on a 4D CBCT performed on the first day of treatment [24]. In addition, a Japanese study by Harada et al. observed a comparable mean volume, but a difference in the maximum amplitudes between tumour motion on the 4DCT and the one performed during radiotherapy [25].

## Conclusions

A personalized PTV margin of 4 mm can be applied for upper lobe lesions located above the carina, if only a single 4DCT is performed. For lower lobe lesions and upper lobe lesions located under the carina, reproducibility of the movement remains uncertain, and a single 4DCT associated to a 3 mm margin can lead to large underdosage to the PTV and ITV.

## Data Availability

The datasets generated during and/or analysed during the current study are available from the corresponding author on reasonable request. This study was registered on « Health Data Hub» and « mesdonnes.unicancer.fr». Treatment data was registered in our institution (No 393).

## Abbreviations

4DCT: Four-dimensional computed tomography
AIO: Orfit all-in-one
AP: Anterior–posterior
GTV: Gross Tumour Volume
ITV: Internal Target Volume
LL: Lower lobe
MIP: Maximum Intensity Projection
PTV: Planning Target Volume
RL: Right-left
RPM: Real-time Position Management
SBRT: Stereotactic body radiation therapy
SI: Superior-inferior
UL: Upper lobe
